# Reducing unnecessary culturing: a systems approach to evaluating urine culture ordering and collection practices among nurses in two acute care settings

**DOI:** 10.1186/s13756-017-0278-9

**Published:** 2018-01-08

**Authors:** Robert Redwood, Mary Jo Knobloch, Daniela C. Pellegrini, Matthew J. Ziegler, Michael Pulia, Nasia Safdar

**Affiliations:** 1 Division of Infectious Disease, Department of Medicine, University of Wisconsin-Madison School of Medicine and Public Health, 750 Highland Ave, Madison, Wisconsin 53705 USA; 2 William S. Middleton Memorial Veterans Hospital, 2500 Overlook Terrace, Madison, Wisconsin 53705 USA; 3 Department of Infectious Disease, University of Chicago Medical Center, 5841 S Maryland Ave, Chicago, Illinois 60637 USA; 4 Division of Infectious Disease, Perelman School of Medicine, University of Pennsylvania, 3400 Civic Center Blvd, Philadelphia, Pennsylvania 19104 USA; 5 BerbeeWalsh Department of Emergency Medicine, University of Wisconsin-Madison School of Medicine and Public Health, 800 University Bay Drive, Madison, Wisconsin 53705 USA

**Keywords:** Acute care, Antibacterial agents, Asymptomatic bacteriuria, Inappropriate prescribing, Prevention & Control, Emergency department, Intensive care unit, Nursing, SEIPS, Urinalysis, Urine culture

## Abstract

**Background:**

Inappropriate ordering and acquisition of urine cultures leads to unnecessary treatment of asymptomatic bacteriuria (ASB). Treatment of ASB contributes to antimicrobial resistance particularly among hospital-acquired organisms. Our objective was to investigate urine culture ordering and collection practices among nurses to identify key system-level and human factor barriers and facilitators that affect optimal ordering and collection practices.

**Methods:**

We conducted two focus groups, one with ED nurses and the other with ICU nurses. Questions were developed using the Systems Engineering Initiative for Patient Safety (SEIPS) framework. We used iterative categorization (directed content analysis followed by summative content analysis) to code and analyze the data both deductively (using SEIPS domains) and inductively (emerging themes).

**Results:**

Factors affecting optimal urine ordering and collection included barriers at the person, process, and task levels. For ED nurses, barriers included patient factors, physician communication, reflex culture protocols, the electronic health record, urinary symptoms, and ED throughput. For ICU nurses, barriers included physician notification of urinalysis results, personal protective equipment, collection technique, patient body habitus, and Foley catheter issues.

**Conclusions:**

We identified multiple potential process barriers to nurse adherence with evidence-based recommendations for ordering and collecting urine cultures in the ICU and ED. A systems approach to identifying barriers and facilitators can be useful to design interventions for improving urine ordering and collection practices.

## Background

Asymptomatic bacteriuria (ASB), also known as an asymptomatic urinary tract infection, is defined as isolation of bacteria in an appropriately collected urine sample from an individual without signs or symptoms referable to the urinary tract [1]. While the prevalence of ASB varies by population, it is a common phenomenon and occurs in 1–5% of healthy adult women and 2–10% of pregnant women. It is highly prevalent in diabetic women (9–27%), nursing home residents (25–50%), and spinal-cord injury patients (23–89%) [1].

The Infectious Disease Society of America (IDSA) recommends against routine screening for ASB (except in pregnant women and patients undergoing urological surgery), rejection of all samples with greater than five squamous epithelial cells per low-power field, and consideration of an alternate diagnosis to UTI in patients without urinary symptoms [2]. Treatment of ASB in most clinical settings is not recommended [3]. In addition, the American Geriatrics Society and the American Board of Internal Medicine recently identified treatment of ASB as an overused intervention in their “Choosing Wisely” campaign [4]. Even with these guidelines, however, recent studies have shown a 20–80% gap between what is recommended and what is practiced [5–7].

Previous studies have identified barriers to appropriate management of ASB with an emphasis on knowledge gaps. Trautner et al. surveyed 169 providers at a tertiary care center and found that decreased knowledge regarding the recognition and management of ASB was associated with poor familiarity with guidelines and cognitive biases (i.e. organism type, patient age, and presence of pyuria influenced the inappropriate use of antibiotics) [7]. Drekonja et al. identified a similar knowledge gap among 100 surveyed resident physicians with 14% reporting that they had read the IDSA guideline on management of ASB and a mean of 37% correct answers when provided with five clinical vignette questions regarding the management of ASB in nonsurgical scenarios [8].

Additional studies have investigated non-sterile urine collection techniques and the unnecessary ordering of urine cultures as upstream factors that contribute to the unnecessary treatment of ASB [9, 10]. However, there is little known regarding the barriers and facilitators that affect nurse initiated ordering and collection practices in the emergency department (ED) and intensive care unit (ICU) where time constraints may increase the risk of over testing or improper collection techniques [11].

We undertook a qualitative study to investigate urine culture ordering and collecting practices among ED and ICU nurses at an academic medical center. Our aim was to identify key systems and human factors that affect concordance with recommended urine ordering and collection practices among nurses. At the time the study was conducted, reflex urine culture testing was in place-urinalyses with pyuria were automatically processed for urine cultures.

## Methods

### Conceptual framework and focus group design

**Fig. 1:**
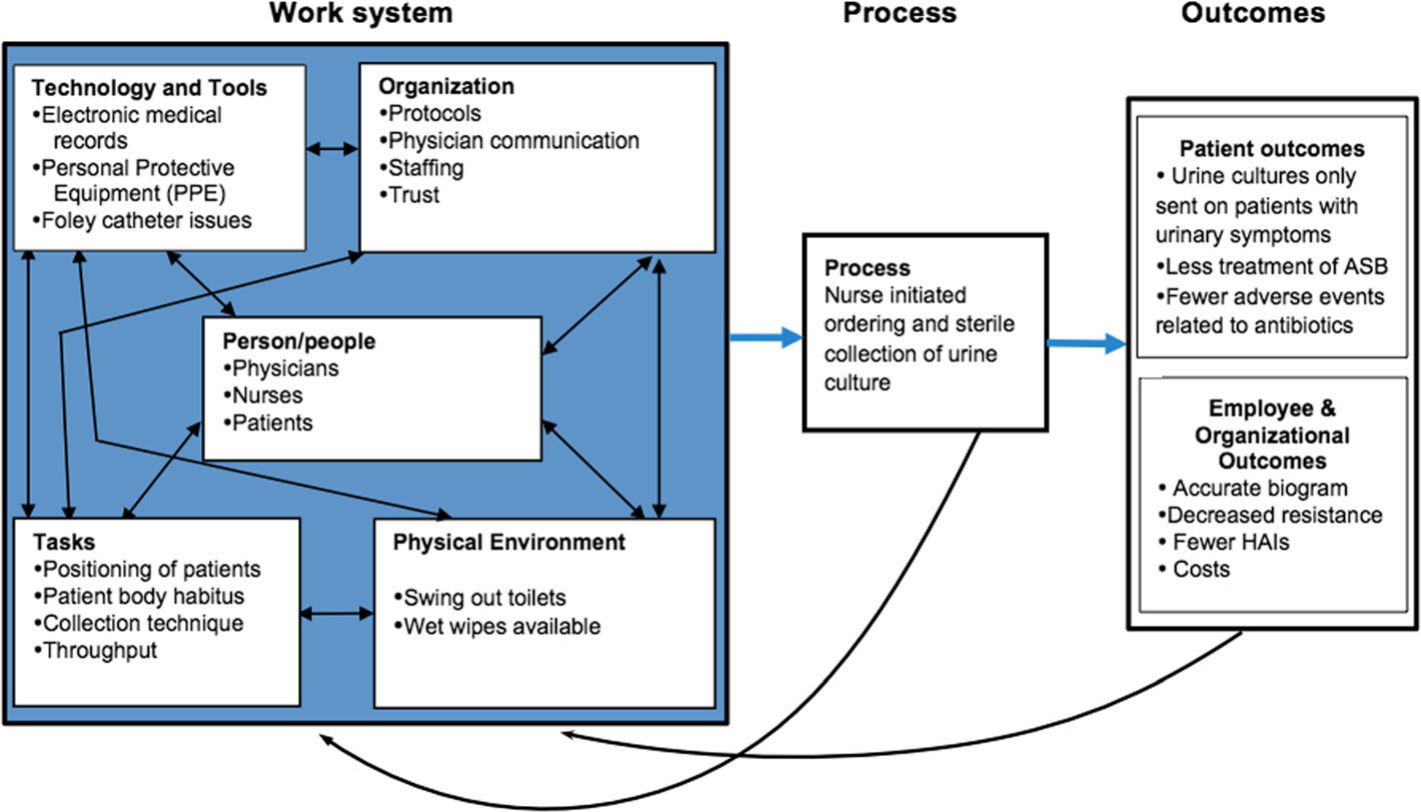
The Systems Engineering Initiative for Patient Safety (SEIPS) model, adapted from Carayon et al. 2006

We conducted focus groups to investigate both the systems and human factors elements that affect urine sample collection and urine collection techniques. ED and ICU nurses were chosen as our subjects of interest because acute care nurses face unique challenges (uncertainty of diagnosis in hemodynamically unstable patients, resuscitation/stabilization concerns, time constraints, and incomplete clinical information). Additionally, antibiotic decisions made in these challenging settings heavily influence subsequent outpatient and inpatient care plans. We used the Systems Engineering Initiative for Patient Safety (SEIPS) model, a framework for understanding structures, processes and outcomes in healthcare to model our focus group questions [12]. The work system elements included in the SEIPS model are person, process, task, organization, tools/technology, physical environment. These elements can affect patient and employee/organizational outcomes, and can be used to improve patient safety. Systems engineering principles are becoming increasingly common for care redesign in acute care settings where teamwork and multi-step care is common [13]. The strengths of this model include its focus on work system design while taking a broad view of care processes and outcomes. Fig. 1 shows the SEIPS model adapted to this study [12].

Two 45-min focus groups were held at our 592-bed tertiary care medical center, between the months of October 2014 and February 2015. Participants were selected by convenience sampling: all nurses in each unit were invited to participate and all who responded were included. We asked a series of open-ended questions regarding system elements impacting urine ordering and collection practices. The distinction between asymptomatic bacteriuria and UTIs was not addressed. Focus groups were audio-recorded and transcribed by authors MZ and DP. Specific questions are listed in Tables 1 and 2. This was a QI project which was deemed exempt and IRB review was not required.

**Table 1:** ED RN focus group questions (SEIPS framework domain in parentheses)

How often do you look at a patient’s urine sample? (Task)
Walk us through the steps of what you would do if you have concerns about a patient’s urine when you are first evaluating them in the ED? (Process)
What challenges are there for collecting an adequate urine sample and how do they effect whether or not you order a urine culture? (Physical environment)
Which guidelines are you aware of for the ordering of urine cultures? (Organization)
What do you think about the communication between nursing and physicians regarding urine culture ordering? (Person)
What clinical presentations other than dysuria would prompt you to think about ordering a urine culture or a urinalysis with reflex culture? (Task)
Do you feel that there is a role for the electronic health record for decision support in terms of when to order a urine culture? (Technology and tools)
How do you feel this issue affects patient safety or quality of care? (Patient outcome)
How do your job demands influence the ordering of urine cultures? (Organization)
How does this topic affect your job satisfaction or stress? (Employee and organizational outcome)

**Table 2:** ICU RN focus group questions (SEIPS framework domain in parentheses)

What is the longest, oldest catheter that has been in one of your patients? (Person)
Could you walk us through the steps of what you would do if you were concerned about a patient’s urine? What prompts you to order a urine culture? (Organization)
How do you collect a urine sample from both a patient who is with and without a catheter or nephrostomy tube? (Task)
Would you say that there are challenges or difficult processes about collecting a clean catch sample compared to doing it with a Foley or nephrostomy tube? (Task)
When do you assess patients for urinary symptoms or changes in urine appearance/output? How does that fit into your work day? (Process)
Suppose you have collected a urine sample or you are concerned about urinary symptoms, what kinds of barriers or challenges have you noticed when communicating these concerns to the provider? (Technology and tools)
Is there anything about the work environment that makes it challenging or affects your ability to get a urine sample? (Physical environment)
Do you feel that there is any difference between experienced nurses vs. younger nurses in terms of how to approach ordering urine cultures? (Person)
How do you feel this issue affects patient safety or quality of care? (Patient outcome)
How does this topic affect your job satisfaction or stress? (Employee or organizational outcome)

### Data analysis

We used iterative categorization for our data analysis, a systematic technique for analyzing qualitative data that is designed to incorporate the pre-selected framework that researchers deem important (in this case the SEIPS framework) and also inductively include emerging themes from the data [14].

We divided analysis into two stages: descriptive and interpretative. In the descriptive stage, we performed line-by-line coding of the interview transcripts using deductive as well as inductive processes. We constrained our deductive coding to the aforementioned SEIPS domains and then counted the number of appearances for each domain and tabulated the results as a frequency plot. During the inductive coding process, we did not constrain the coding by a framework and instead assigned a free-text theme to each statement or segment of the transcript. We then tabulated this more expansive set of inductive coding themes as a unique frequency plot. Two researchers (RR and MJK) performed the coding and we compared agreement between the researchers using Cohen’s kappa statistic for inter-rater reliability. We combined coding results from RR and MJK and averaged the results to yield a set of four preliminary frequency plots. For both the inductive and deductive frequency plots, we considered the top 50% of themes to be dominant themes and selected those for further analysis. Having thus completed the descriptive stage of the analysis, we were left with four final frequency plots, each narrowed to the top 50% of themes: (1) ED Nurses-Deductive, (2) ICU Nurses-Deductive, (3) ED Nurses-Inductive, and (4) ICU Nurses-Inductive.

In the interpretation stage of the analysis, we combined the two inductive frequency plots to identify shared themes that acute care nurses face regarding ordering urine cultures and sterile collection techniques and then did the same for the deductive frequency plots. We present the results both quantitatively as the percentage of the interview content dedicated to each theme and qualitatively as central concepts with representative quotations.

## Results

Participants included five ED nurses and six ICU nurses. All participants were white females with a bachelor degree in nursing, ages ranged from 24 to 54 years old and practice experience ranged from 2 to 25 years. The average weighted Cohen’s kappa statistic for inter-rater reliability was 0.70 for the deductive coding, 0.91 for the inductive coding, and 0.81 overall. This indicates fair agreement between the two researchers for deductive coding, excellent agreement for inductive coding, and good agreement overall (Table 3).

**Table 3:** Inter-rater reliability results for each focus group transcript

	κ Statistic	95% CI	*p*-value
ED RN Inductive	0.890	0.7612–0.9180	0.02
ICU RN Inductive	0.931	0.8650–0.9967	0.04
ED RN Deductive (SEIPS framework)	0.641	0.4421–0.8399	<0.05
ICU RN Deductive (SEIPS framework)	0.772	0.5774–0.8999	0.04

### Deductive results (combined ED and ICU nurses)

Systems-based coding results (structured, deductive) were similar for both ED and ICU nurses with person (25% ED, 30% ICU), process (24% ED, 26% ICU), and task (17% ED, 17% ICU) comprising the dominant themes. For example, one ICU nurse expressed that rarely do physicians not trust her judgment: “If I say, ‘boy that [urine] really smells and you should check that out’, the provider will say ‘ok’ and they will write the [urinalysis with urine culture] order.” This quotation is a representative ICU nurse comment that was coded under “person” [nurse-physician dyad] in the deductive analysis. Similarly, the quotation, “Nurses will have a patient void in a hat and then will collect that as a UA when that really isn’t the best sample because that really isn’t sterile,” was an ED nurse comment that was coded under “process” [collection process] and “I feel that the urine [in Foley catheters] looks suspicious for infection” was an ED nurse comment that was coded under “task” [interpretation of urine appearance]. Table 4 contains a list of dominant themes paired with illustrative quotations.

**Table 4:** Deductive Results: Dominant Themes and Illustrative Quotations

Dominant Theme	Illustrative Quotation
(Percent of focus group content pertaining to SEIPS framework domain)	
Person (25% ED, 30% ICU)	“Finding the physician [is a challenge to obtaining a urinalysis order]” –ED RN A “Rarely do [the physicians] not trust me. If I say boy that [urine] really smells and you should check that out, the provider will say ok and they will write the [urinalysis with urine culture] order.” –ICU RN A
Process (24% ED, 26% ICU)	“Nurses will have a patient void in a hat and then will collect that as a UA when that really isn’t the best sample because that really isn’t sterile.” –ED RN D “[Nurses] who are new will ask someone who is more senior, if they would ask the doctor for an order if a urine [sample] looked weird.” –ICU RN F
Task (17% ED, 17% ICU)	“I feel that the urine [in Foley catheters] looks suspicious for infection.” “It always does.” –ED RNs D & E “If it’s a change in the [Foley catheter urine appearance] and [the patient] is communicative and they haven’t complained of anything, I probably would just get the sample before I would think to ask if they were having any urinary symptoms.” –ICU RN F

### Inductive results

In terms of human factors (inductive process), dominant themes for ED nurses included patient factors (13%), physician communication/availability (12%), reflex culture protocols (9%), the electronic health record (7%), urinary symptoms (6%), and ED throughput (5%). For example, the quotation “basically every geriatric patient should get a urinalysis. I’m actually being kind of serious” is a representative ED nurse comment that was coded under “patient factors” [geriatric patients] in the inductive analysis.

Dominant human factor themes for ICU nurses included physician notification of urinalysis results (24%), personal protective equipment (13%), collection technique (13%), patient body habitus (12%), and Foley catheter issues (12%). For example, the quotation “if I were straight cathing [sic] a large person I probably would gown up to protect my arms since you are going to be in there” is a representative ICU nurse comment that was coded under “personal protective equipment” [protective gown facilitating proper collection technique] in the inductive analysis.

Tables 5 and 6 contain a full list of dominant themes paired with illustrative quotations for ED and ICU focus groups, respectively.

**Table 5:** ED Nurse Inductive Results: Dominant Themes and Illustrative Quotations

Dominant Theme	Illustrative Quotation
(Percent of focus group content pertaining to theme)	
Patient Traits (13%)	“Basically every geriatric patient should get a urinalysis. I’m actually being kind of serious.” –ED RN E “[Patients need to have] the supplies to clean themselves adequately because that little piece of paper…really? The people that come down sometimes definitely have some issues that they need to deal with before they give us a sample.” –ED RN C
Physician Communication and Availability (12%)	“If we think the urine looks really nasty and then I’ll go ahead and add the [urine culture] order.” –ED RN D “There wasn’t communication [between nurses and physicians]; the order would just go in and the physician would see it.” –ED RN B
Reflex Culture Protocol (9%)	“We did not order [isolated] urine cultures; it was always reflex urinalysis with culture” –ED RN C
Electronic Health Record (7%)	“That’s annoying when [a clinical care reminder] pops up; it’s already being addressed.” –ED RN E “I think that in my head I say, ‘that’s the protocol,’ but I don’t actually go into the computer to review it” –ED RN B
Symptoms (6%)	“I always thought that it was lab that looked at the results; said that there the white blood cells were up and then setup the culture. It wasn’t my decision or the physician’s decision, or patient presentation that drove that, rather objective data.” –ED RN D
Throughput (5%)	“People call back the next day and say that the urine was clean while they were in the ER but then their culture grew bacteria, so what about those people? They say that ‘[my] primary care doctor called me and told me that I need to be put on antibiotics.’” –ED RN A

**Table 6:** ICU Nurse Inductive Results: Dominant Themes and Illustrative Quotations

Dominant Theme	Illustrative Quotation
(Percent of focus group content pertaining to theme)	
Physician Notification of Urinalysis Results (24%)	“If you have a new patient and [the physicians] are questioning what kind of sepsis they were having, then I would be more cognizant of if there was bacteria or white blood cells in the patient’s urine. But otherwise, not really.” –ICU RN E
Personal Protective Equipment (13%)	“If I were straight cathing a large person I probably would gown up to protect my arms since you are going to be in there. If you are going to be doing something that’s fluidy, I would still wear them because they are outside of the rooms, whether they are ordered or not.” –ICU RN F (on PPE being a facilitator of proper straight catheterization technique)
Collection Technique/Clean Catch (13%)	“If you are going to get a straight cath [sample] sterilely for a female, at least, you need two nurses, regardless of their size.” “Right, and then if it’s a bigger person, then it can take five to six potentially.” –ICU RNs E & C
Patient Body Habitus (12%)	“Trying to get a clean catch on patients who don’t mobilize and can’t stand over a toilet, that is [an environmental barrier] too because we don’t really have bathrooms, we have swing out toilets that are too low for most of our patients anyways.” –ICU RN F
Foley Catheter (12%)	“If you are super swamped in another room and a nurse helps you by dumping your Foley bag, [they may help you out by saying], ‘hey, did you notice that your urine looks terrible?’” –ICU RN D

## Discussion

We identified multiple potential process barriers to nurse compliance with evidence-based recommendations for ordering and collecting urine cultures in the ICU and ED. Process facilitators (i.e. technology, materials, work systems, people that help the process) were rarely mentioned and questions designed to extract information about facilitators were typically answered with comments about barriers.

Within the work system, barriers related to the acute care environment at the person, process, and task level tended to dominate the conversation. The emphasis on nurses’ difficulty in locating physicians to discuss results (person) and nurse-initiated ordering protocols that default to a urinalysis with reflex culture (process) demonstrates how communication breakdown can lead to work-arounds or sub-optimal practice. Similarly, the emphasis on patient body habitus compromising sterile collection technique or expectations of rapid collection (tasks) underscores how the pace of work can lead to skipping key steps in favor of work-flow efficiency. It is not surprising that ED and ICU nurses identified similar system-level barriers since both environments are fast-paced and rely heavily on both nurse autonomy and teamwork between nurses and physicians. In healthcare, teamwork can create checks and balances that contribute to patient safety, but autonomy can promote efficiency. For example, ideal care may be for a nurse and physician to take a moment and discuss together whether a patient requires a urine culture, which may be challenging to achieve with high fidelity [15]. Similarly, obtaining a truly sterile straight catheter urine sample on an obese patient may require more resources in personnel time than are readily available. In such situations, there may be a trade-off between autonomy over teamwork essentially compromising one care process (sterile technique) in favor of another (efficiency). Increasing our understanding of the competing priorities that health care providers may face in the complex system of healthcare organizations is an essential step in optimizing high reliability systems that promote best practices.

The following findings from the inductive analysis merit attention: First, nurses in both settings displayed either a misinterpretation or a desire for more knowledge in terms of when it is recommended to order a urine culture. Common misconceptions were that—in the absence of symptoms—dark or foul-smelling urine, change in Foley catheter urine appearance, or presence of bacteruria/pyuria justified sending a urine culture. One ED nurse also identified a system design flaw in which unnecessary urine cultures were sent from the ED and primary care physicians would recommend antibiotics for their patients a few days later at follow up based on positive culture results [16]. This nurse observation mirrors a larger trend that has been identified in the inpatient literature; namely that 61% of admitted patients with positive urine cultures may have ASB [17, 18]. System interventions like requiring a guideline-concordant indication for ordering of urine cultures (as protocol) may enhance adherence to national guidelines. Person/environment-level interventions such as targeted education campaigns or guideline pocket cards in the department can complement system level interventions [19].

Second, the ED nurse cohort called attention to physician communication as a barrier. This attributed to increased time constraints in the ED (rapid patient turnover and throughput) and lack of standardized electronic communication tools (as opposed to the ICU nurses who had a universally-used pager system in place to communicate with the physicians). ED nurses also had urinalysis ordering protocols in place that provided more autonomy in terms of nurse-initiated ordering than the ICU protocols. Rapid ED throughput is essential to patient safety, since ED crowding is a known risk factor for medical error and delays of care [20]. Nevertheless, communication issues are often solvable without drastic compromises in throughput. Examples of system interventions include a team huddle (as protocol) at various times throughout an ED shift or the inclusion of an antibiotic stewardship item on the departmental rounds checklist. Person/tools-level interventions could include staff cell phones or “post-it” messages in the electronic health record to facilitate rapid communication of changes in patient status or urinalysis results, even when providers are not in close proximity [21].

Third, we found that a trusting relationship between physicians and nurses may paradoxically pose a barrier to appropriate urine culture ordering. While trust between providers is certainly a positive part of effective teamwork, trust without shared understanding can result in miscommunication and medical error. In our focus groups, some nurses were not familiar with the IDSA guidelines that urine cultures should typically only be ordered in the presence of urinary symptoms, but then went on to voice that physicians trust them to order cultures using their judgement, including when patients are asymptomatic but the urine appears cloudy or had a foul smell. These knowledge gaps may often go unnoticed and interventions that address these gaps are needed.

Fourth, we found that challenges with following proper urine culture collection techniques existed in both clinical settings, but varied based on patient factors. In the ED, the primary concern was that patients did not use pre-collection cleansing kits in the proper manner, thus providing contaminated samples. Rather than slow down patient movement through the ED by re-sending a clean catheter sample, providers sometimes sent cultures on contaminated samples. In the ICU, sedated patients or obese patients were often a barrier to collecting a proper sample, although nurses did note that PPE was a facilitator of proper collection techniques; primarily, because they felt more comfortable cleansing the patient’s perineum when they were protected from skin and body fluid contact. This was particularly true in the most obese patients, where straight catheter collection required close physical contact. Our understanding of interventions to improve sterile urine collection continues to evolve and interventions that emphasize patient education or standardized cleansing techniques have produced equivocal results [22]. Further research in this area is needed.

Fifth, ED nurses found it was inconvenient to look up urine culture protocols and guidelines in the electronic health record and, in one instance, commented that the alerts associated with sending inappropriate cultures were a nuisance. These observations underscore a larger systems issue; namely, alert fatigue and its ability to compromise the utility of clinical decision support systems [23]. One illustrative example of a solution for alert fatigue was proposed by Rush et al. who found that having a multidisciplinary committee audit the electronic health record to minimize low-yield alerts helped to ensure that the clinical decision support system enhances, rather than hinders patient care [24].

Sixth, both groups stated that older patients and patients with an indwelling Foley catheter were more likely to have urine cultures sent. Since these two groups often suffer from cognitive impairment and deficits in sensation, they are high-risk for diagnostic uncertainty and inappropriate treatment of ASB and may represent a high-yield starting point for targeted interventions. One low-cost intervention that is supported in the long-term care literature is the use of an ASB algorithm for older patients and patients with an indwelling catheter. Loeb et al. (2005) provide an effective algorithm for long term care environments that has not been validated in acute care settings [25].

Our literature search revealed two studies that addressed this issue. Jones et al. (2016) surveyed 394 RNs from five hospitals regarding appropriate reasons and methods to obtain urine culture specimens on patients with indwelling Foley catheters [11]. They found that 58% of RNs reported that their colleagues follow recommended collection techniques when collecting urine samples in patients with an indwelling catheter. In March 2017, Fakih and Khatib emphasized in a letter to the editor that “improving the culture of culturing should be viewed as an integral component of antimicrobial stewardship [and] is likely to encourage clinicians to use their clinical judgment in their patient evaluations and to move from a reflexive process to a more reflective one, leading to better care” [26]. They recommended a two-pronged approach to eliminating CAUTIs that includes optimizing care pathways and educating frontline providers on best practices.

We found two studies that specifically investigated nurse initiated ordering and collection practices in a general patient population. Richards et al. (2012) found that replacing a routine urinalysis and culture protocol for all patients in a urology clinic with a nurse practitioner-initiated protocol resulted in a 10% drop in the number of urinalyses and cultures ordered with no change in clinical management [27]. Frazee et al. (2012) found that 61% of 129 surveyed female ED patients received proper midstream parted-labia urinalysis catch instructions from their nurses and 82% of the cohort that received instructions actually understood them [28].

Limitations of this study include small sample size and results derived from a single institution. Convenience sampling of participants also creates a risk of selection bias. Potential disadvantages of using focus groups rather than interviews in this study included disagreements among participants, irrelevant side discussions, and participants feeling under pressure to agree with the dominant view. While we found several misperceptions among nurses related to ordering of the urine cultures, it may be such misperceptions occur among other members of the treating team. We did not conduct focus groups for other members of the treating team in this study as our main focus was on the collection and ordering of urine specimens-a task performed most often by nurses at our institution.

Our findings have implications for infection preventionists and clinicians in the ICU and the ED. When analyzing the complexity of ordering urine culture and urine collection, those working in EDs and ICUs should consider a system level approach that identifies the work system barriers and facilitators to compliance with guidelines. Our study findings can assist in guiding targeted interventions within these acute care settings.

## Conclusions

In this study, we found barriers to appropriate ordering and collection of urine cultures, using a human factors framework. These included common knowledge misconceptions, communication barriers, unfamiliarity with IDSA guidelines/hospital protocols, and a perception that urine cultures were more likely needed in those with a foley catheter or of older age. Our findings may be used to identify interventions to address these barriers and improve quality of care.

## Abbreviations

ASB: Asymptomatic bacteriuria
ED: Emergency department
ICU: Intensive care unit
IDSA: The Infectious Disease Society of America
SEIPS: Systems engineering initiative for patient safety
